# Supplementary material for: Root traits and belowground herbivores relate to plant–soil feedback variation among congeners
Source: Nat Commun. 2019 Apr 5;10:1564. doi: 10.1038/s41467-019-09615-x (PMC6450911; doi:10.1038/s41467-019-09615-x)
Supplement: Supplementary file 1 — Supplementary Information [file 41467_2019_9615_MOESM1_ESM.pdf]

Supplementary information

Root traits and belowground herbivores relate to plant-soil feedback variation among congeners

Wilschut et al.

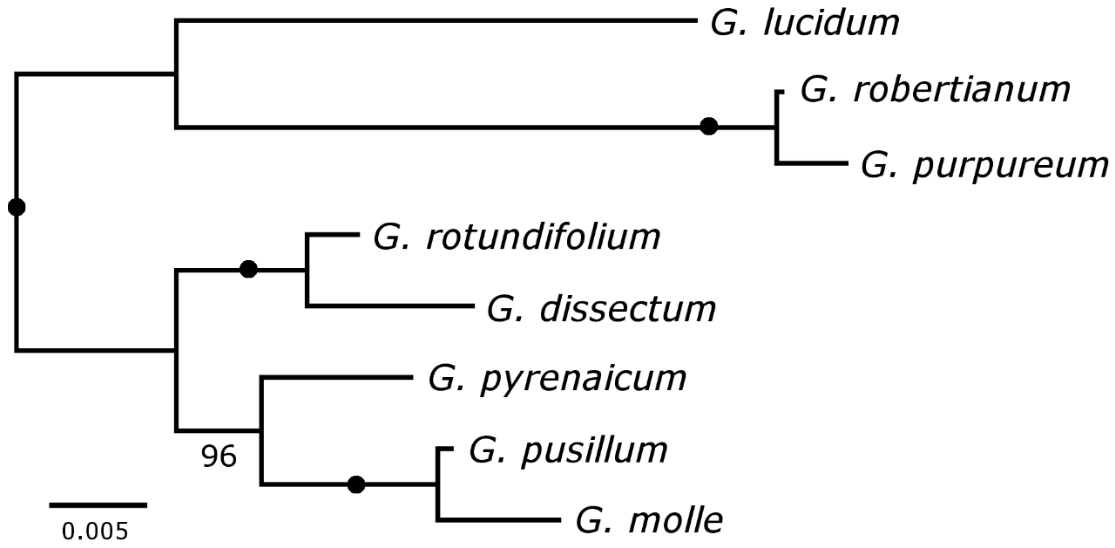

Supplementary Figure 1: Phylogeny of eight *Geranium* species. Phylogenetic relationships among native plant species *G. dissectum, G. molle, G. pusillum* and *G. robertianum,* and range-expanding plant species *G. lucidum*, *G. pyrenaicum*, *G. purpureum* and *G. rotundifolium* (*ROT*), based on DNA-regions rbcL, the trnL gene and the trnL-trnF intergeneric spacer. Bootstrap support is visualized with black dots (100% support) or numbers indicating the percentage support.

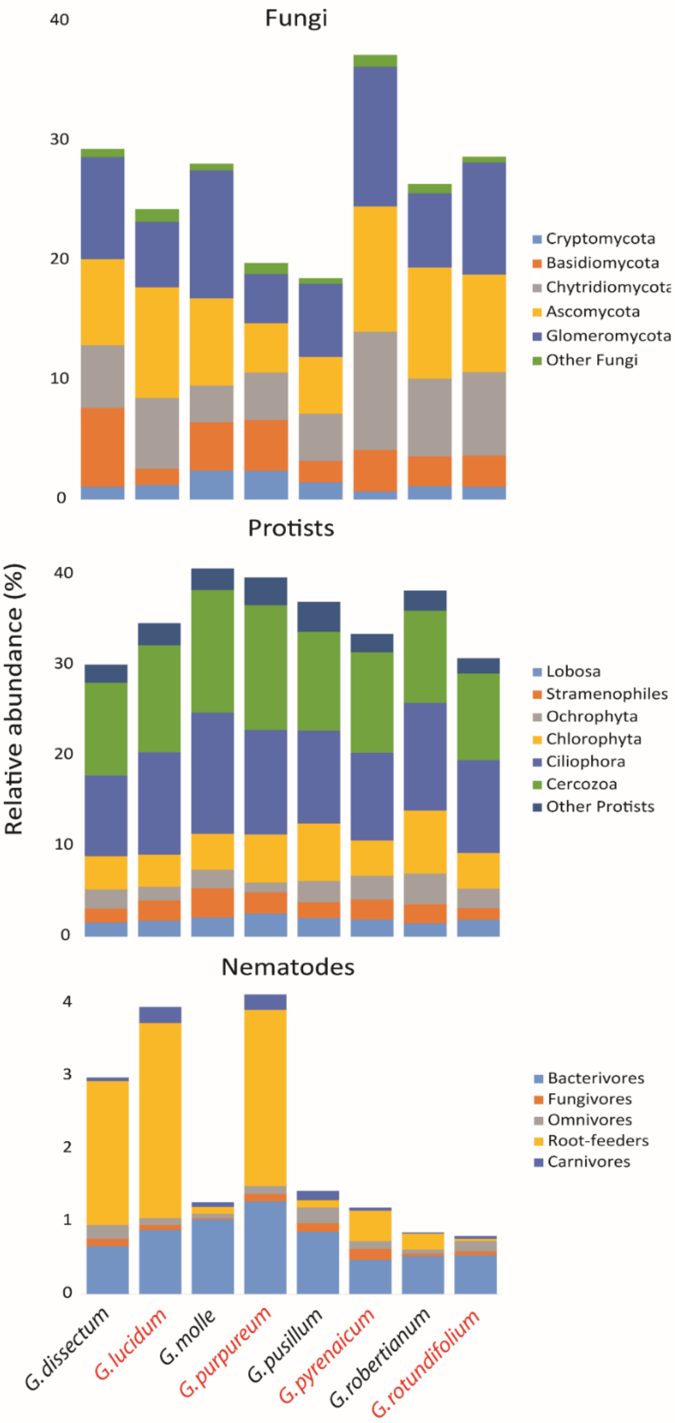

Supplementary Figure 2: Relative abundances eukaryotic groups. Average relative abundances (% 18S rRNA gene reads) of taxonomic groups of fungi and protists and functional groups of nematodes in the rhizospheres of native (black) and range-expanding (red) *Geranium* species.

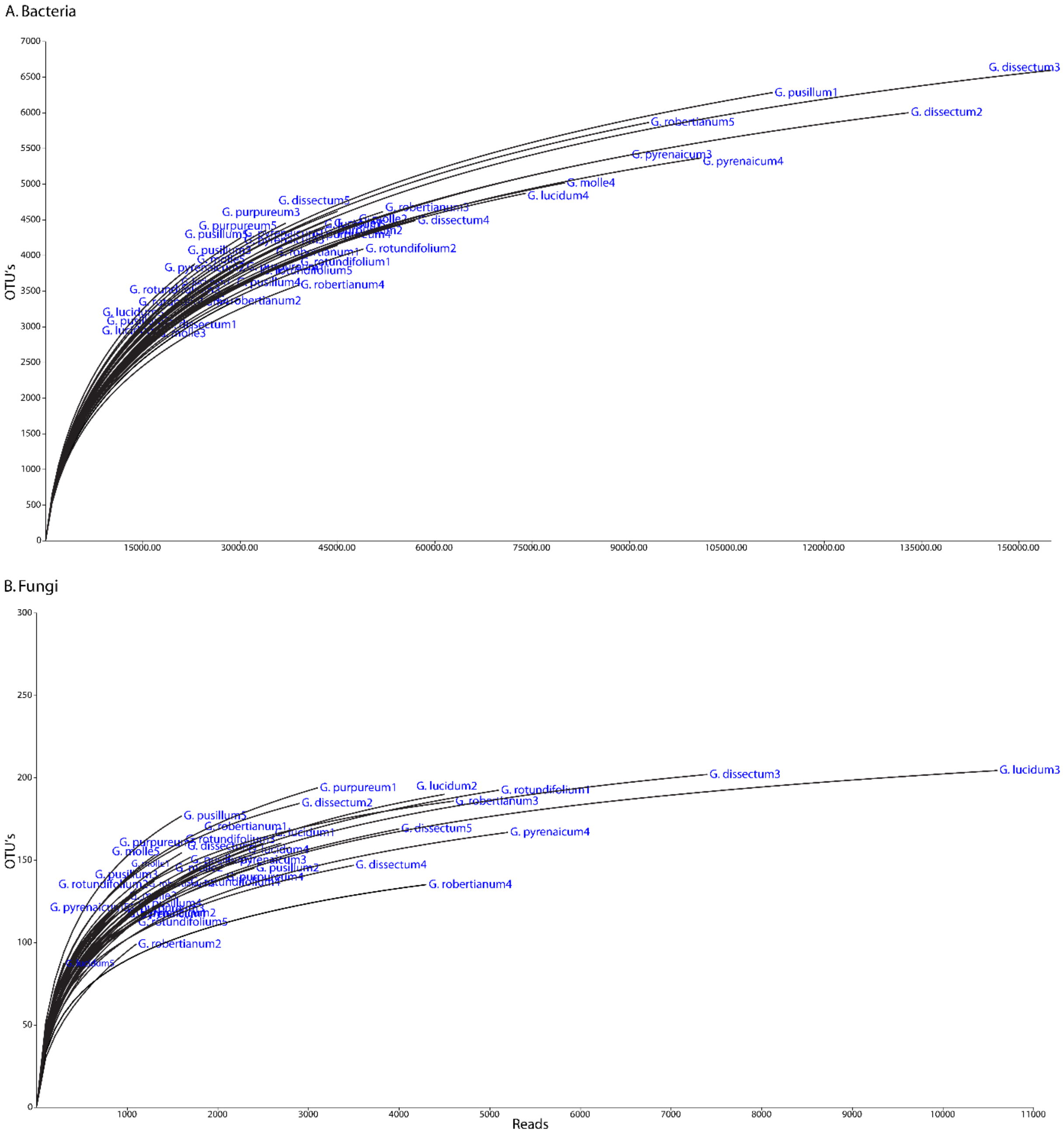
A. Bacteria
OTU's
7000
6500
6000
5500
5000
4500
4000
3500
3000
2500
2000
1500
1000
500
0
15000.00
30000.00
45000.00
60000.00
75000.00
90000.00
105000.00
120000.00
135000.00
150000.00
G. dissectum3
G. pusillum1
G. dissectum2
G. robertianum5
G. pyrenaicum3
G. pyrenaicum4
G. molle4
G. lucidum4
G. dissectum5
G. purpureum3
G. robertianum3
G. dissectum4
G. purpureum5
G. rotundifolium2
G. rotundifolium1
G. rotundifolium5
G. robertianum4
G. robertianum2
G. molle3
B. Fungi
OTU's
300
250
200
150
100
50
0
1000
2000
3000
4000
5000
6000
7000
8000
9000
10000
11000
Reads
G. dissectum3
G. lucidum3
G. purpureum1
G. lucidum2
G. rotundifolium1
G. dissectum2
G. robertianum3
G. pusillum5
G. robertianum1
G. dissectum5
G. pyrenaicum4
G. molle5
G. dissectum4
G. robertianum4
G. pusillum3
G. rotundifolium5
G. robertianum2

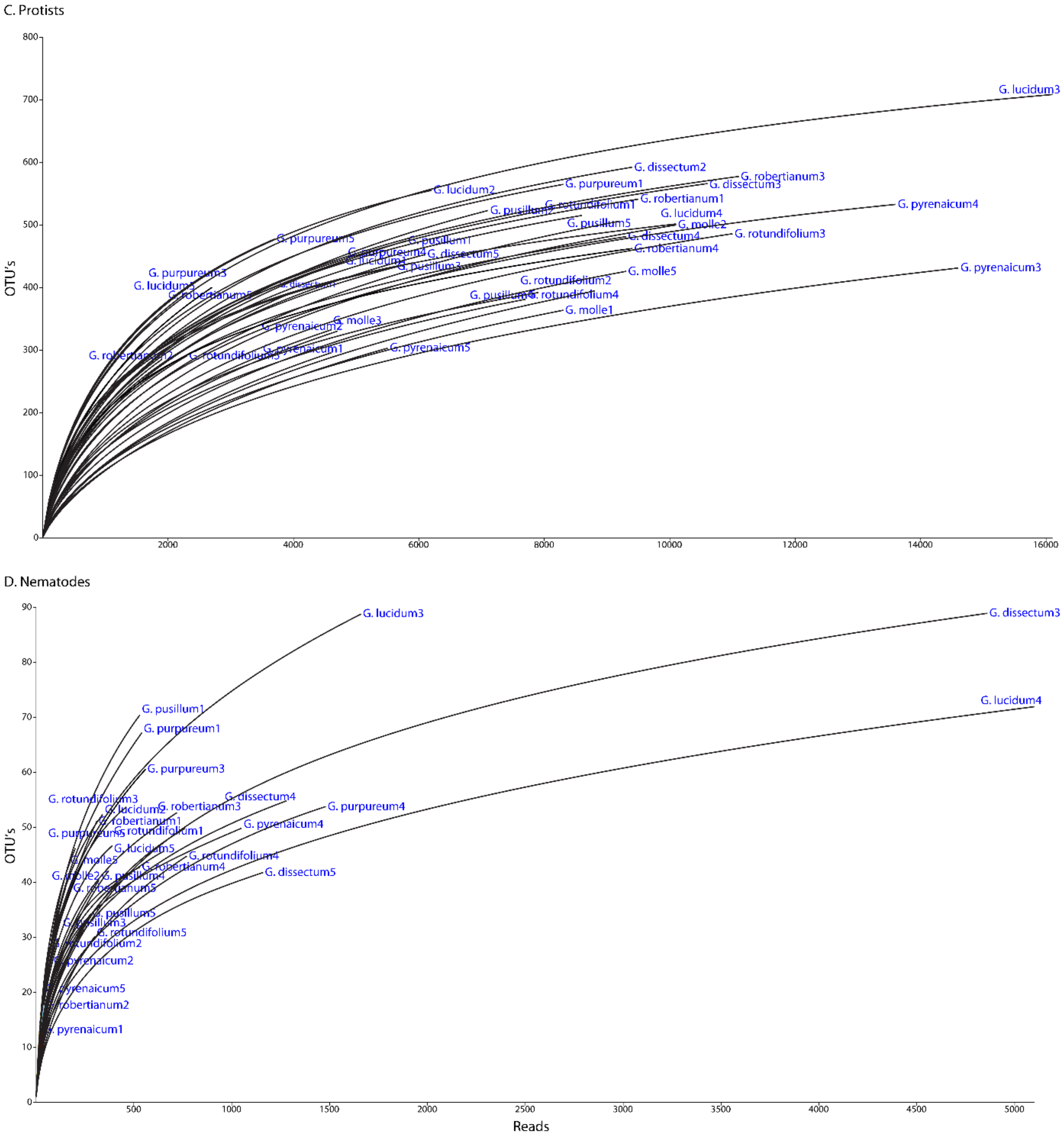

Supplementary Figure 3: Rarefaction curves of major rhizosphere groups. Per-sample relationship between number of reads and OTU-diversity for Bacteria (A), Fungi (B), Protists (C) and Nematodes (D).

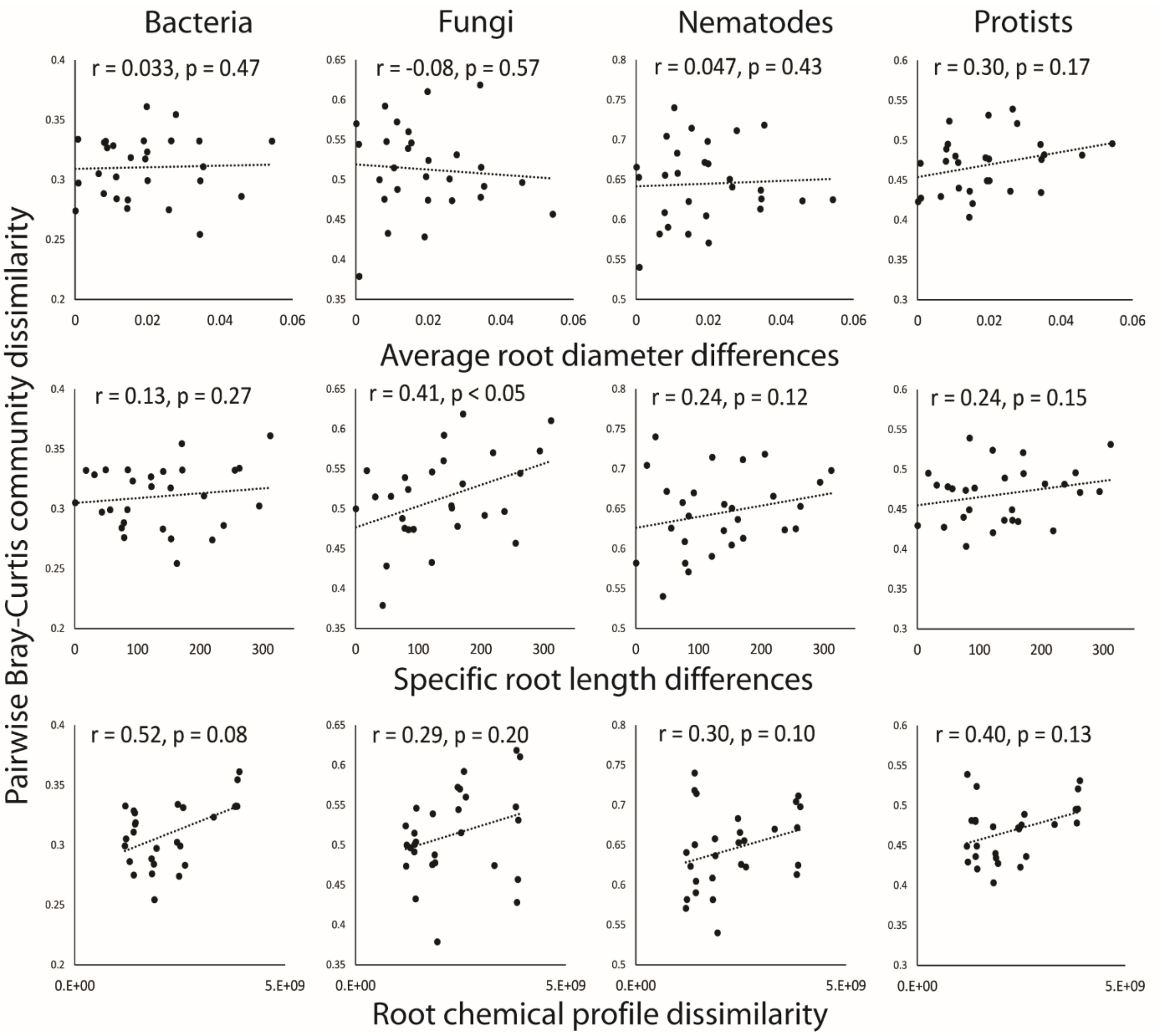

Supplementary Figure 4: Trait-community linkages. Scatterplots of between-species root trait dissimilarities (root chemical profile, specific root length and average root diameter) and Bray-Curtis community dissimilarities (Bacteria, Fungi, Nematode, Protists; also see Fig. 2). R^2 and p-values based on Mantel tests are shown.

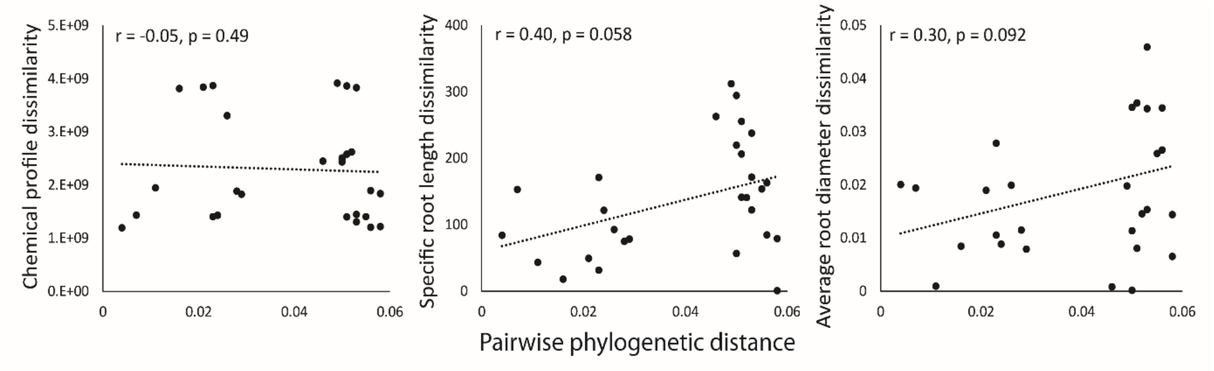

Supplementary Figure 5: Phylogenetic distances and trait dissimilarities. Scatterplots of pairwise phylogenetic distance and root trait dissimilarity (root chemical profile, specific root length and average root diameter). R^2 and p-values based on Mantel tests are shown.

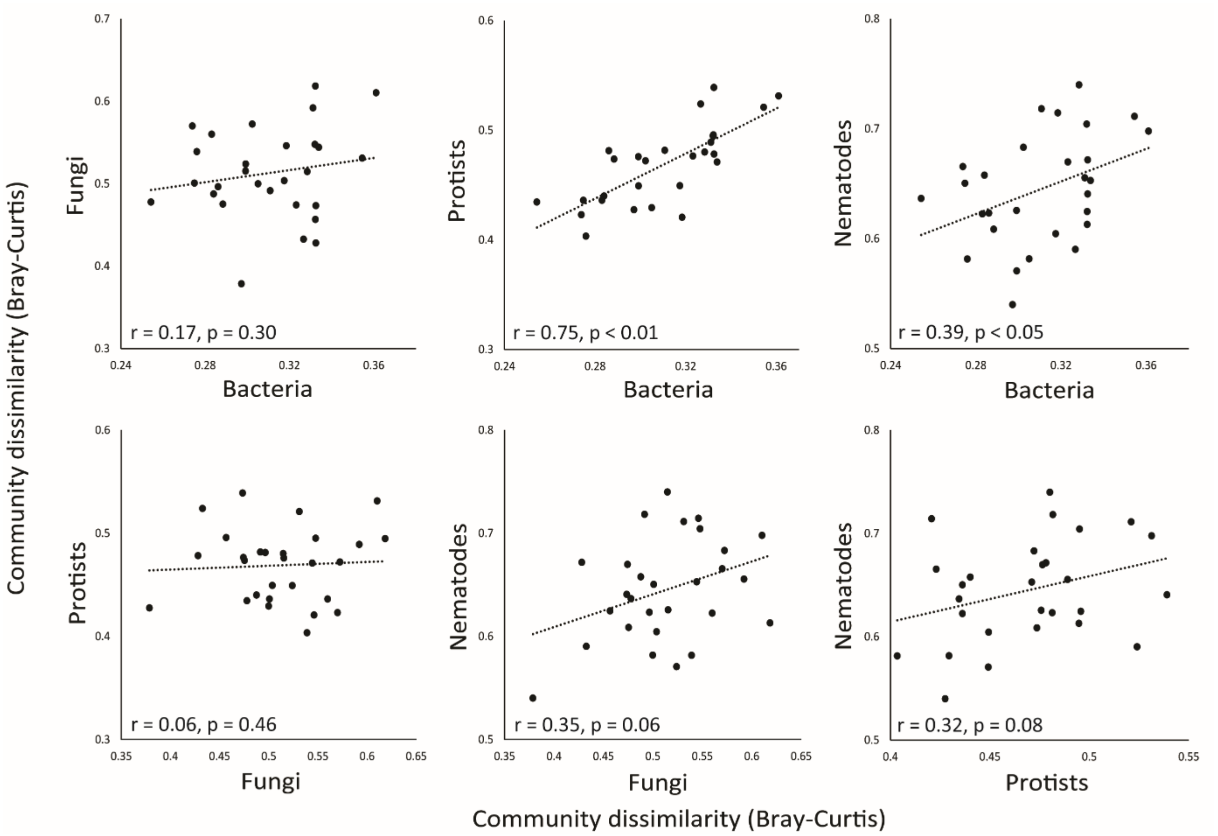

Supplementary Figure 6: Linkages between major rhizosphere groups. Scatterplots between between-species Bray-Curtis community dissimilarities of Bacteria, Fungi, Nematodes and Protists; also see Fig. 2). R^2 and p-values based on Mantel tests are shown.

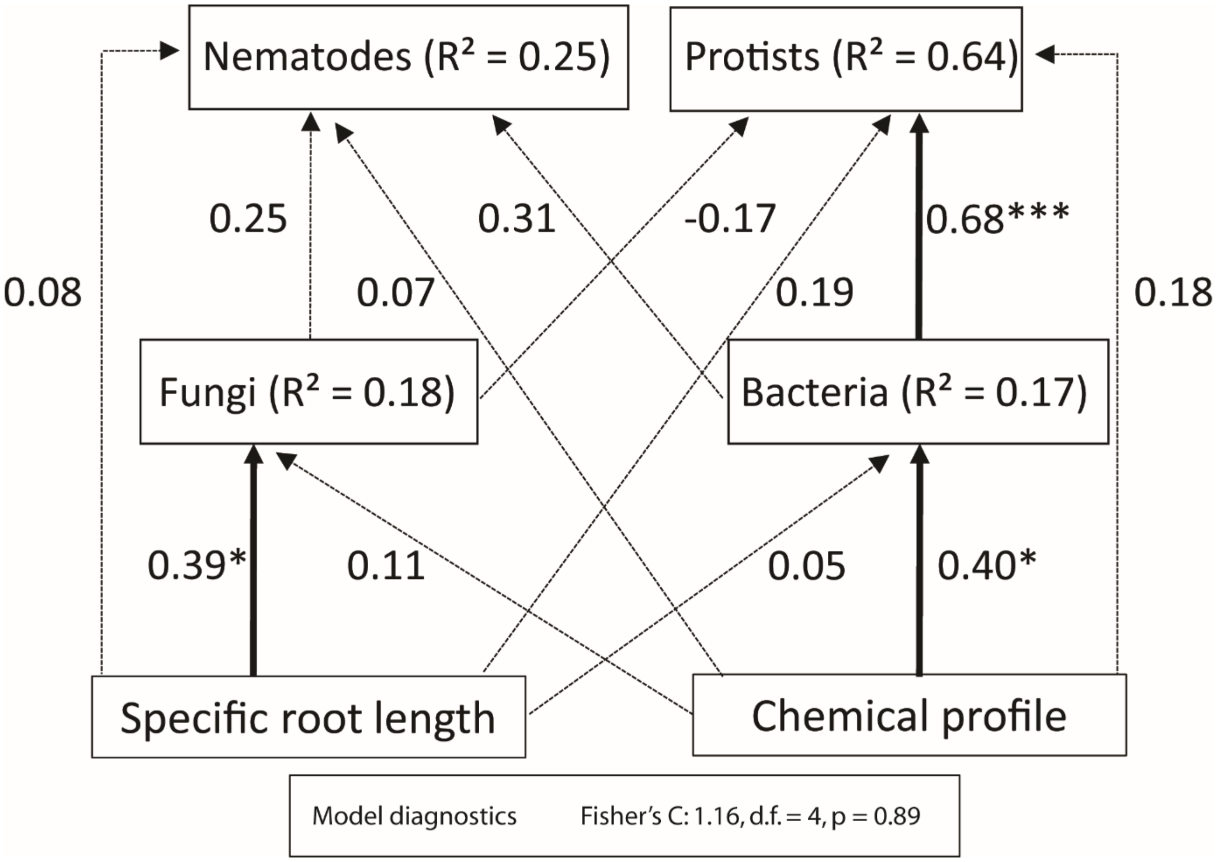

Supplementary Figure 7: Structural equation model on root trait-community linkages. Arrows represent the predictive effects of between-species dissimilarities in root traits (Root chemical profile, Specific root length) on between-species Bray-Curtis dissimilarity in rhizosphere community groups (Bacteria, Fungi, Protists, Nematodes), and predictive effects of between-species dissimilarities in fungal and bacterial community composition on dissimilarities in nematode and protist community composition. Bold and dashed arrows respectively represent significant and non-significant predictive relationships. Model estimates of predictor strength, and explained variation (R^2) of the community groups are shown.

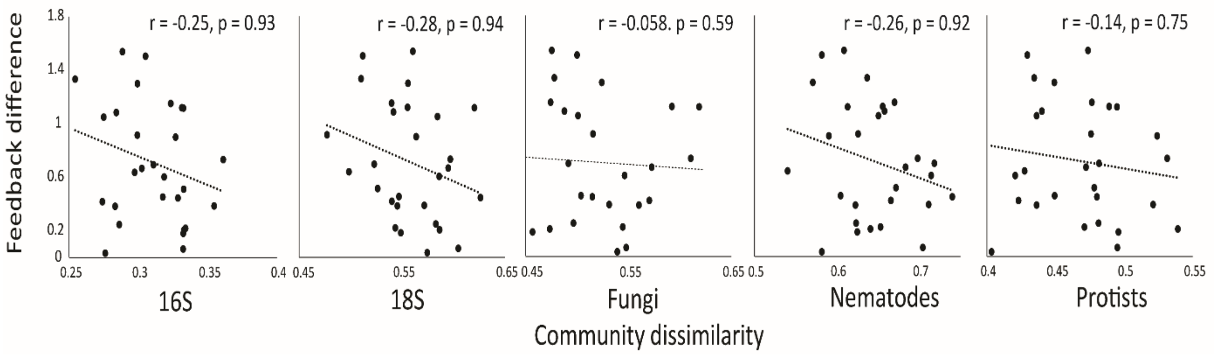

Supplementary Figure 8: Community dissimilarity and plant-soil feedback variation. Scatterplots between Bray-Curtis community dissimilarities (16S rRNA gene, 18S rRNA gene, Fungi, Nematode, Protists) and plant-soil feedback differences show no positive association between community dissimilarity of different *Geranium* species and their pairwise differences in plant-soil feedback. Pearson correlation coefficients r and p-values of Mantel-tests are shown.

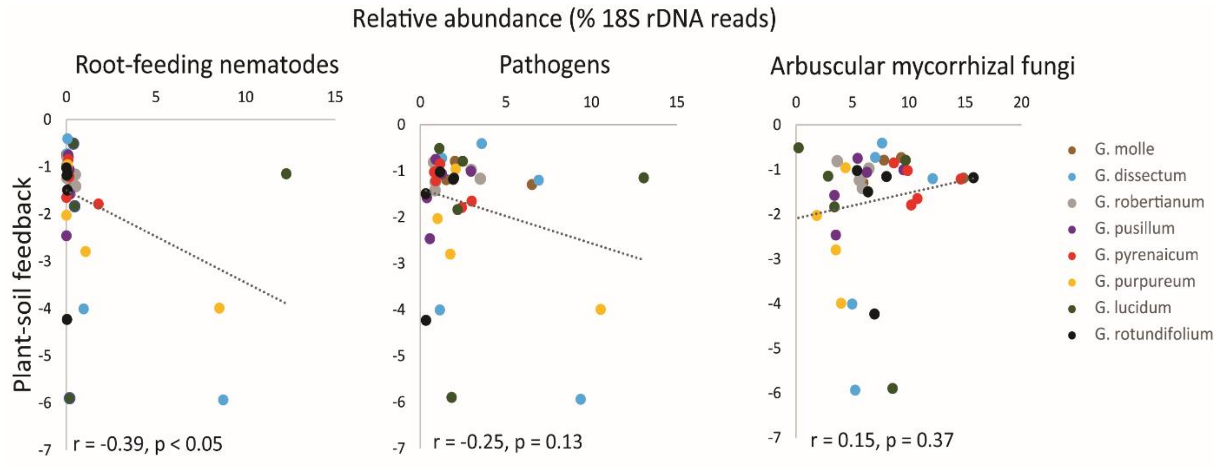

Supplementary Figure 9: Predictors of plant-soil feedback. Correlations between individual plant-soil feedback replicates (ln($biomass_{conditioned}$/$biomass_{control}$) of eight *Geranium* species (5 replicates per species; see Fig. 4 for averaged values) and the relative abundances (% 18S rRNA gene reads) of root-feeding nematodes, plant pathogens (see Methods) and arbuscular mycorrhizal fungi (AMF) in the conditioned soils. Pearson correlation coefficients r and p-values of Pearson correlation tests are shown.

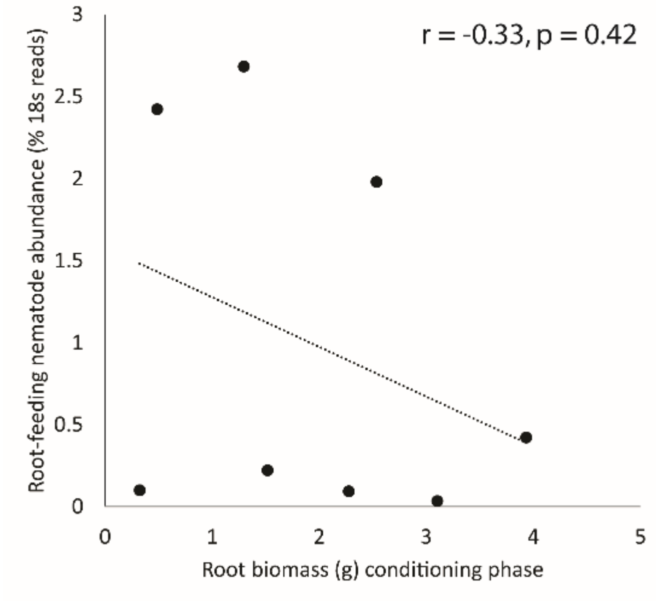

Supplementary Figure 10: Root-feeding nematode accumulation and root biomass in conditioning phase. Scatterplot between average plant root biomass and the relative abundance of root-feeding nematodes at the end of the soil conditioning phase shows that high relative abundances of root-feeding nematodes are not associated with high root biomass of plants in the conditioning phase. Pearson correlation coëfficient and p-value of the Pearson correlation test are shown.

Supplementary Table 1: Predictors of rhizosphere community composition. Plant species and plant origin effects on the composition of the components of the *Geranium* rhizosphere communities, and results of Mantel test on the correlations between pairwise phylogenetic distances and pairwise community dissimilarity among the eight *Geranium* species.

	**Plant species**		**Plant origin**		**Phylogeny**
	Expl. Var. (RDA)	Permutation test	Expl. Var. (RDA)	Permutation test	Mantel cor, p
**16S (all OTUs)**	23.2%	Pseudo-F: 1.2, p<0.01	3.1%	Pseudo-F: 1.1, p = 0.22	-0.23, p = 0.84
**18S (all OTUs)**	24.5%	Pseudo-F: 1.2, p<0.01	3.2%	Pseudo-F: 1.1, p = 0.27	0.04, p = 0.42
**Fungi**	26.2%	Pseudo-F: 1.3, p<0.01	3.4%	Pseudo-F: 1.1, p = 0.21	0.39, p < 0.05
**Protists**	23.9%	Pseudo-F: 1.2, p<0.01	3.1%	Pseudo-F: 1.0, p = 0.44	-0.09, p = 0.63
**Nematodes**	28.2%	Pseudo-F: 1.5, p<0.01	3.8%	Pseudo-F: 1.3, p = 0.16	0.10, p = 0.31

Supplementary Table 2: Origin of *Geranium* seeds used in the present study. Latitude and longitudes, as well as municipality names of all the collection sites. All sites are located in The Netherlands.

**Species**	**Location population**	**Latitude**	**Longitude**
Geranium pyrenaicum	Groesbeek	51.778294	5.92699
Geranium molle	Wageningen	51.959453	5.65838
Geranium dissectum	Wageningen	51.959453	5.65838
Geranium pusillum	Wageningen	51.960901	5.655526
Geranium rotundifolium	Wageningen	51.965554	5.67321
Geranium purpureum	Leiden	52.14441	4.499412
Geranium robertianum	Wageningen	51.965897	5.677823
Geranium lucidum	Amersfoort	52.178606	5.38548

Supplementary Table 3: Ellenberg indicator values for eight *Geranium* species. Ellenberg values (scaled from 1 to 9) indicate species preferences for light, moisture, pH and nitrogen conditions (based on: Hill *et al* (1999)). Conditions associated with the values are given in brackets (low numbers-high numbers).

	Light (shaded-light)	Moisture (dry-wet)	Reaction (acidic-alkaline)	Nitrogen (low-high)
G. dissectum	7	5	7	6
G. lucidum	6	4	7	6
G. molle	7	5	6	5
G. purpureum	7	3	6	3
G. pusillum	7	4	7	7
G. pyrenaicum	8	4	7	6
G. robertianum	5	6	6	6
G. rotundifolium	7	4	7	6

Supplementary Table 4: Collection locations of independent replicate soils. Latitude and longitudes, as well as municipality names of all the collection sites of the 5 soil inocula, used as independent replicates in the plant-soil feedback experiment.

Soil replicate	Location	Latitude	Longitude
1	Rhenen	51.95284	5.580714
2	Rhenen	51.95383	5.607912
3	Wageningen	51.9595	5.658224
4	Wageningen	51.96475	5.681148
5	Renkum	51.97078	5.736912

Supplementary Table 5: Overview of replicate numbers for rhizosphere community sequencing. Total number of successfully sequenced replicate communities are shown for sequencing of eukaryotes (18S rRNA gene) and prokaryotes (16S rRNA gene).

Species	Replicates 18S	Replicates 16S
Geranium dissectum	5	5
Geranium lucidum	5	5
Geranium molle	4	5
Geranium purpureum	4	5
Geranium pusillum	5	5
Geranium pyrenaicum	5	5
Geranium robertianum	5	5
Geranium rotundifolium	5	5

Supplementary Table 6: Functional group assignment. Overview of microbial and nematode taxa assigned to functional groups (root-feeding nematodes, plant pathogens, arbuscular mycorrhizal fungi). Numbers of OTU's of each taxa are shown.

Functional group	Taxon	Number of OTU's
Root-feeding nematodes	*Boleodorus*	1
Root-feeding nematodes	*Meloidogyne*	3
Root-feeding nematodes	*Mesocriconema*	1
Fungal plant pathogen	*Rhizoctonia*	1
Protist plant pathogen	*Pythium*	3
Protist plant pathogen	*Haptoglossa*	1
Protist plant pathogen	Oomycota unspecified	8
Protist plant pathogen	*Polymyxa*	3
Protist plant pathogen	*Spongospora*	3
Protist plant pathogen	Plasmodiophorida unspecified	2
Arbuscular mycorrhizal fungi	*Glomeromycota unspecified*	15
Arbuscular mycorrhizal fungi	*Glomus*	21
Arbuscular mycorrhizal fungi	*Ambispora*	1
Arbuscular mycorrhizal fungi	*Diversispora*	1
