# Supplementary material for: Root traits and belowground herbivores relate to plant–soil feedback variation among congeners
Source: Nat Commun. 2019 Apr 5;10:1564. doi: 10.1038/s41467-019-09615-x (PMC6450911; doi:10.1038/s41467-019-09615-x)
Supplement: Supplementary file 3 — Reporting Summary [file 41467_2019_9615_MOESM3_ESM.pdf]

natureresearch

Corresponding author(s): Rutger Wilschut

Reporting Summary

Nature Research wishes to improve the reproducibility of the work that we publish. This form provides structure for consistency and transparency in reporting. For further information on Nature Research policies, see Authors & Referees and the Editorial Policy Checklist.

Statistical parameters

When statistical analyses are reported, confirm that the following items are present in the relevant location (e.g. figure legend, table legend, main text, or Methods section).

n/a	Confirmed	
☐	☒	The exact sample size (n) for each experimental group/condition, given as a discrete number and unit of measurement
☐	☒	An indication of whether measurements were taken from distinct samples or whether the same sample was measured repeatedly
☐	☒	The statistical test(s) used AND whether they are one- or two-sided<br>*Only common tests should be described solely by name; describe more complex techniques in the Methods section.*
☒	☐	A description of all covariates tested
☒	☐	A description of any assumptions or corrections, such as tests of normality and adjustment for multiple comparisons
☐	☒	A full description of the statistics including central tendency (e.g. means) or other basic estimates (e.g. regression coefficient) AND variation (e.g. standard deviation) or associated estimates of uncertainty (e.g. confidence intervals)
☐	☒	For null hypothesis testing, the test statistic (e.g. F, t, r) with confidence intervals, effect sizes, degrees of freedom and P value noted<br>*Give P values as exact values whenever suitable.*
☒	☐	For Bayesian analysis, information on the choice of priors and Markov chain Monte Carlo settings
☒	☐	For hierarchical and complex designs, identification of the appropriate level for tests and full reporting of outcomes
☐	☒	Estimates of effect sizes (e.g. Cohen's d, Pearson's r), indicating how they were calculated
☐	☒	Clearly defined error bars<br>*State explicitly what error bars represent (e.g. SD, SE, CI)*

Our web collection on statistics for biologists may be useful.

Software and code

Policy information about availability of computer code

Data collection	All code for statistics is available from packages described below. Code of the Hydra pipeline used for sequence curation is available (nioo-knaw/hydra: 1.3.3 (Version 1.3.3) (Zenodo, 2017).)
Data analysis	We used Canoco 5 for multivariate analyses, r-packages vegan (for community dissimilarity measurements, mantel tests), lm and lsmeans (general linear model on plant biomass), piecewiseSEM (structural equation model), mass and phia (generalized linear model nematode reproduction). MetaboAnalyst 3.0 was used for chemometric analyses.

For manuscripts utilizing custom algorithms or software that are central to the research but not yet described in published literature, software must be made available to editors/reviewers upon request. We strongly encourage code deposition in a community repository (e.g. GitHub). See the Nature Research guidelines for submitting code & software for further information.

Data

Policy information about availability of data

All manuscripts must include a data availability statement. This statement should provide the following information, where applicable:

- Accession codes, unique identifiers, or web links for publicly available datasets
- A list of figures that have associated raw data
- A description of any restrictions on data availability

Provide your data availability statement here.

Field-specific reporting

Please select the best fit for your research. If you are not sure, read the appropriate sections before making your selection.

☐ Life sciences ☐ Behavioural & social sciences ☒ Ecological, evolutionary & environmental sciences

For a reference copy of the document with all sections, see nature.com/authors/policies/ReportingSummary-flat.pdf

Ecological, evolutionary & environmental sciences study design

All studies must disclose on these points even when the disclosure is negative.

Study description	The core study is a plant-soil feedback experiment, in which 8 plant species individually condition soil in 5 mesocosms (40 mesocosms total). These 5 mesocosms represent 5 independent soils, which were used for all plant species. As a control, mesocosms with the same soils, without plants, were established. After conditioning, Myseq Illumina sequencing was used to identify the soil community in conditioned soils. Thereafter, new individuals of the same plant species were planted in each individual soil, also control soils, to examine feedback responses. A different pot experiment was used to grow plants in sterilized soil to determine structural root atraits, and another experiment was used to grow plants for examination of root chemistry (DART-analysis). We used Genbank to extract DNA-sequences for plant phylogeny reconstruction. Then we integrated these experiments by testing correlations between phylogeny, traits, soil community composition and plant-soil feedback.
Research sample	8 species from the plant genus Geranium, all native to Europe: Geranium molle, Geranium dissectum, Geranium robertianum, Geranium pussillum, Geranium lucidum, Geranium purpureum, Geranium pyrenaicum and Geranium rotundifolium. The latter four are range-expanding plant species and recently colonized areas in northern Europe. This study system provides the unique opportunity to test the effects of nativeness and phylogenetic distance among congeneric plant species.
Sampling strategy	Our study is largely based on a pot experiment with 8 plant species, each with five replicates per treatment; we used 5 different soils as independent replicates: a normal sample size for greenhouse mesocosm experiments.
Data collection	Data was recorded by the first author (plant-soil feedback experiment), the third author (DART-analysis) and the fifth author (nematode reproduction and structural plant traits). Plant biomass data (for plant-soil feedback estimates) was collected and recorded by separating, drying and weighing shoots and roots. Rhizosphere community data was obtained after DNA-extraction (by first, fourth and last author), PCR's (first and seventh author) and sequencing (BGI, China). Structural root trait data was obtained by the 5th author, using biomass measurements and a root scanner. Root chemistry analyses were obtained using DART-measurements, performed at Wageningen University, by first and third author.
Timing and spatial scale	A 14 week conditioning phase for the feedback experiment was used to allow a strong conditioning of soil communities; 7 weeks of plant growth in the feedback phase was sufficient to examine growth responses, while plants did not suffer from pot limitation. The length of the nematode reproduction experiment (12 weeks) allowed at least three cycles of reproduction.
Data exclusions	For the calculations of community dissimilarity and relative abundances of functional groups in the rhizosphere, we excluded 2 samples for which less than 1000 18S reads were available. This cut-off was used so that good estimation of the eukaryotic community composition was ensured. We excluded one replicate of the nematode reproduction experiment, in which no nematodes were found - highly likely a result of an inoculation error.
Reproducibility	We did not repeat our experiment, but used (1) independent replicate soils to increase the variation in our plant-soil feedback experiment and (2) experimentally verified the correlation between plant-soil feedback by means of a nematode reproduction experiment.
Randomization	Our plant-soil feedback experiment, as well as the nematode reproduction experiment were set-up as randomized block experiments. Each block contained a different soil. Blocks were situated next to each other in a greenhouse compartment. As such, variation between blocks could also represent small environmental differences within the greenhouse. Within blocks, mesocosms were randomized.
Blinding	We did not apply blinding in the allocation of seedlings to different treatments, but ensured the use of seedlings of the same size in all experiments.

Did the study involve field work? ☐ Yes ☒ No

Reporting for specific materials, systems and methods

Materials & experimental systems

n/a	Involved in the study
☒	☐ Unique biological materials
☒	☐ Antibodies
☒	☐ Eukaryotic cell lines
☒	☐ Palaeontology
☒	☐ Animals and other organisms
☒	☐ Human research participants

Methods

n/a	Involved in the study
☒	☐ ChIP-seq
☒	☐ Flow cytometry
☒	☐ MRI-based neuroimaging
